# Supplementary material for: Effect of Increased Ionic Liquid Uptake via Thermal Annealing on Mechanical Properties of Polyimide-Poly(ethylene glycol) Segmented Block Copolymer Membranes
Source: Molecules. 2021 Apr 8;26(8):2143. doi: 10.3390/molecules26082143 (PMC8068311; doi:10.3390/molecules26082143)
Supplement: Supplementary file 1 [file molecules-26-02143-s001.pdf]

Supplementary File

Effect of Increased Ionic Liquid Uptake via Thermal Annealing on Mechanical Properties of Polyimide-Poly(ethylene glycol) Segmented Block Copolymer Membranes

Gokcen A. Ciftcioglu[a,b,*], Curtis W. Frank[b]

[a]Department of Chemical Engineering, Marmara University, Istanbul, 34722, Turkey
[b]Department of Chemical Engineering, Stanford University, Stanford, CA 94305, USA

FTIR Spectra of PAAs and PEMs

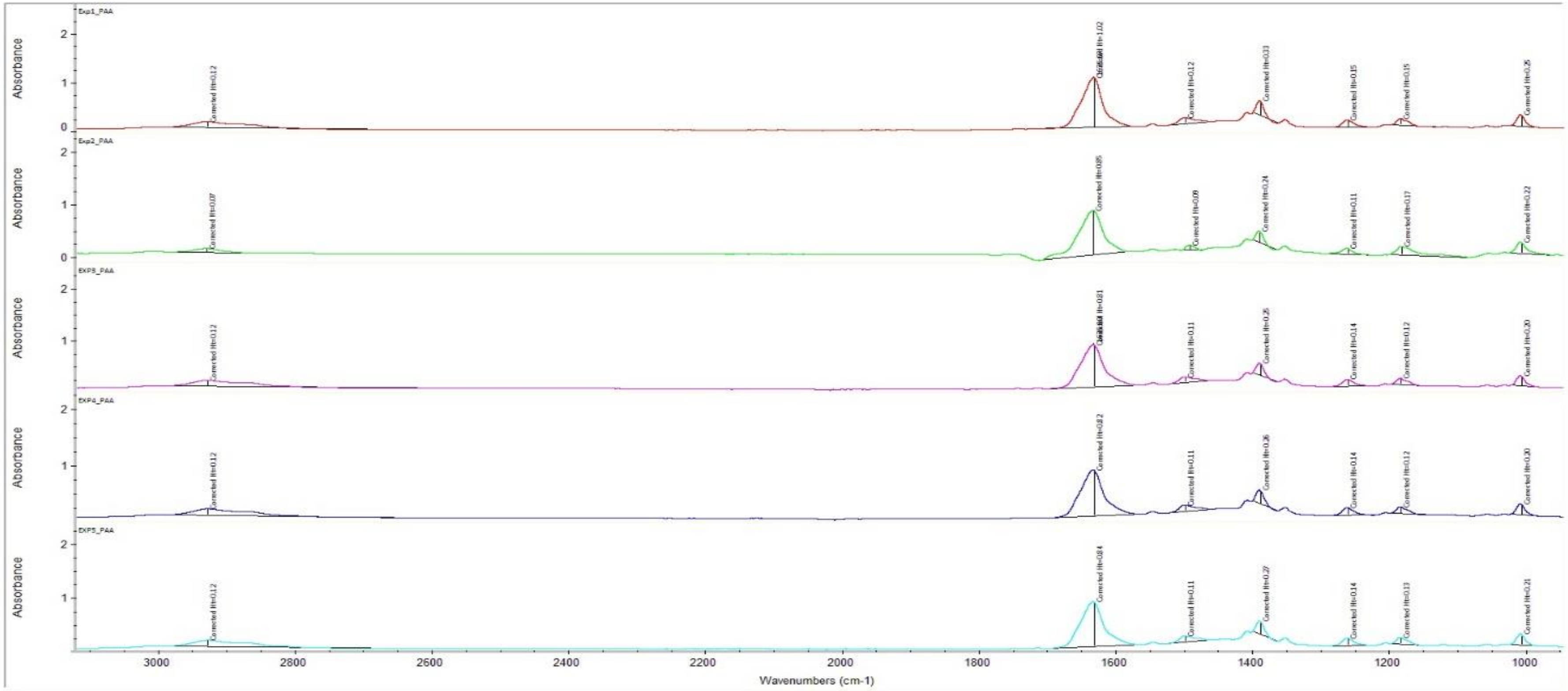

Supp. Fig 1. FTIR specta of PAAs 1-5 (used diamine is PDODA) and height data of the peaks

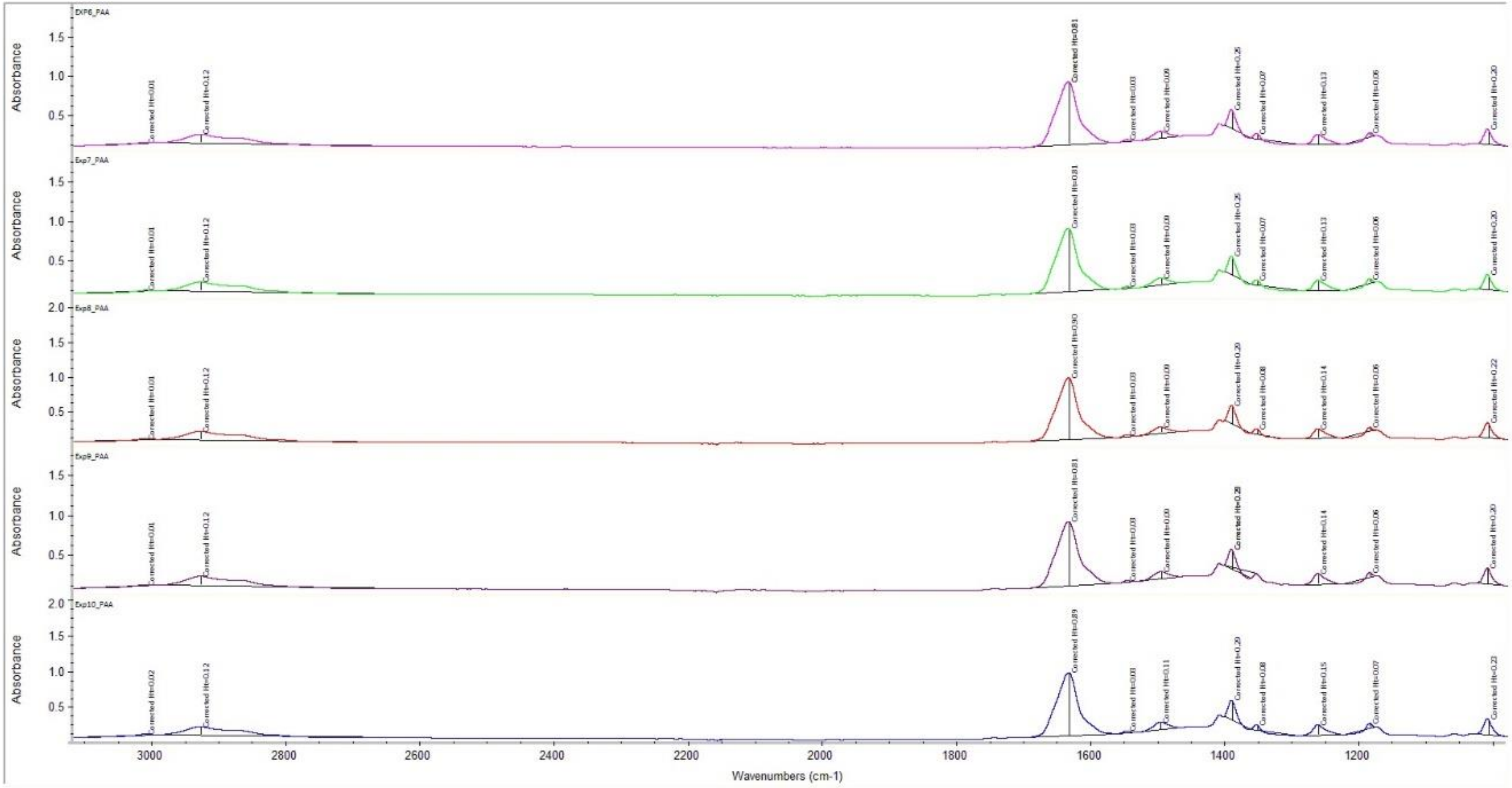

Supp. Fig 2. FTIR specta of PAAs 6-10 (used diamine is AP6F) and height data of the peaks

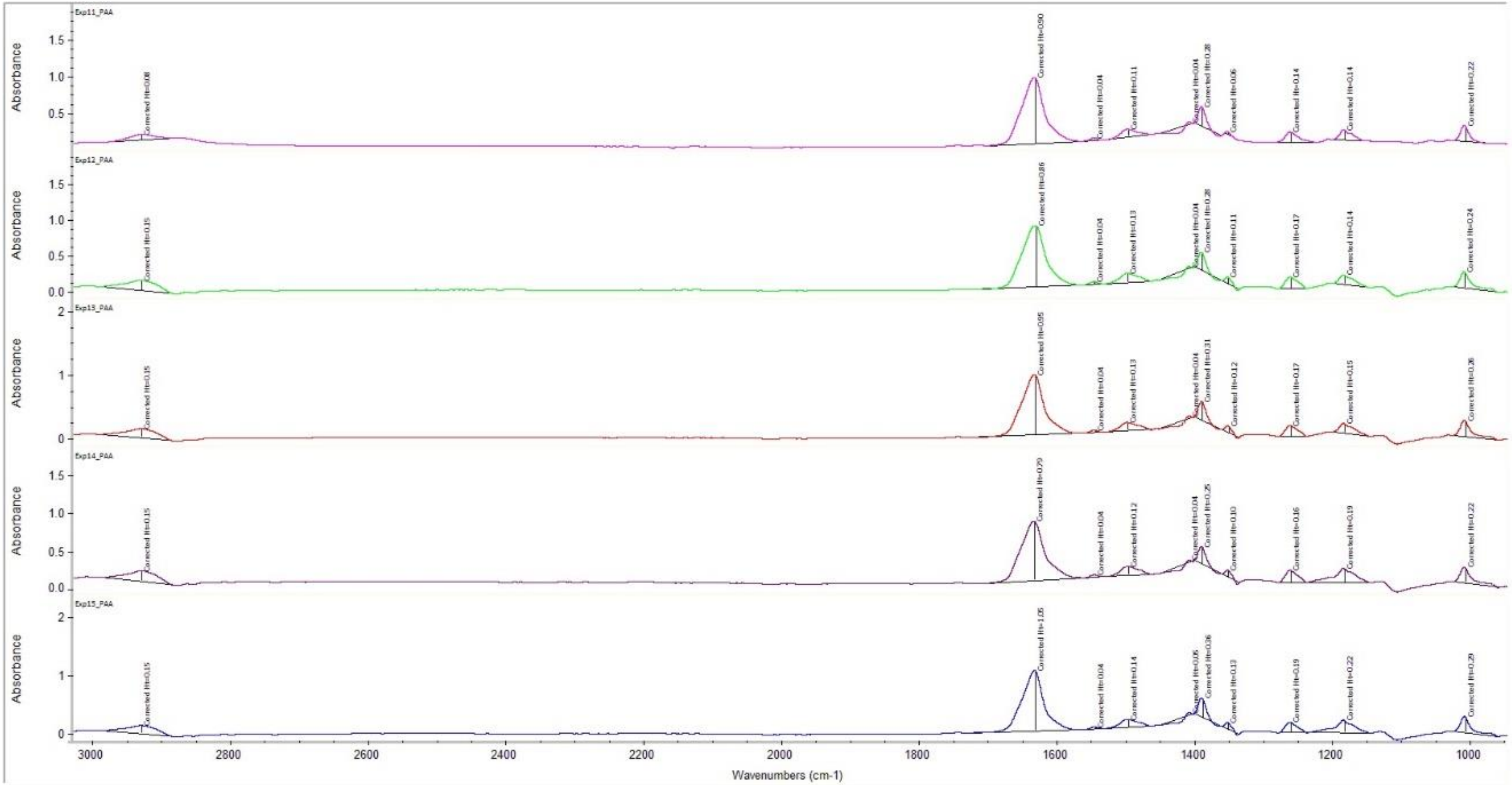

Supp. Fig 3. FTIR specta of PAAs 11-15 (used diamine is equal amounts of PDODA and AP6F) and height data of the peaks

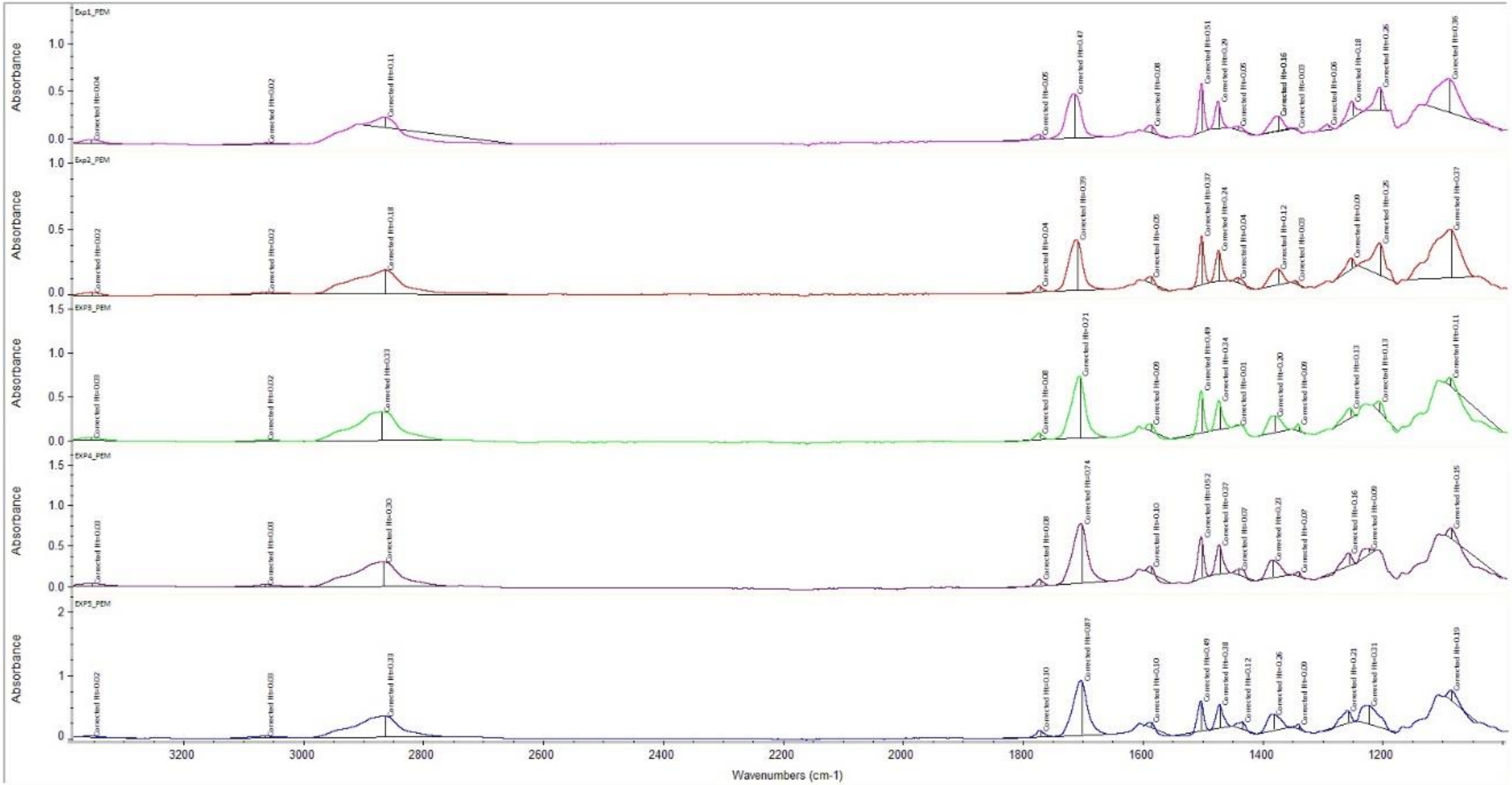

Supp. Fig 4. FTIR specta of PEMs 1-5 (used diamine is PDODA) and height data of the peaks

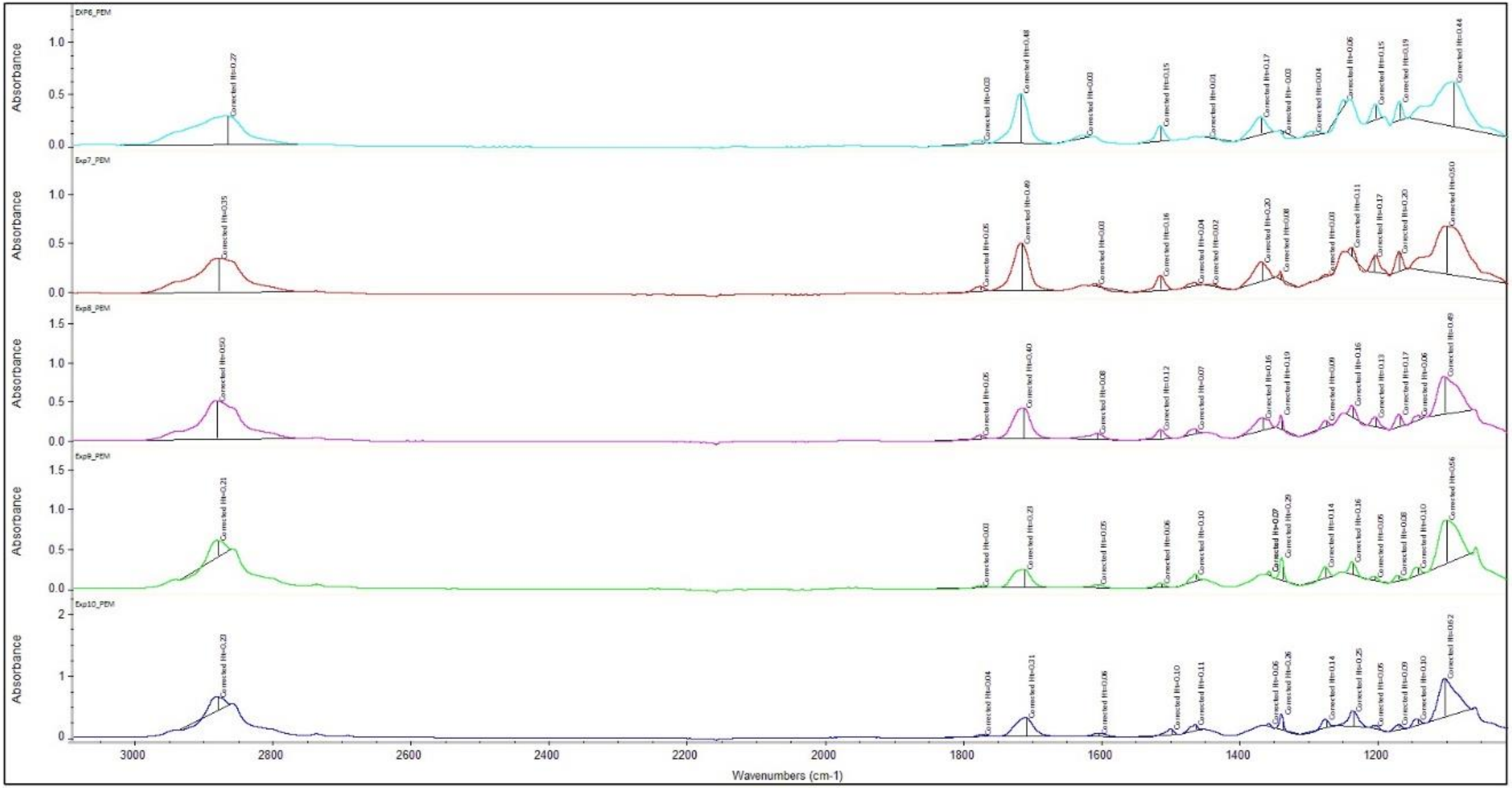

Supp. Fig 5. FTIR specta of PEMs 6-10 (used diamine is AP6F) and height data of the peaks

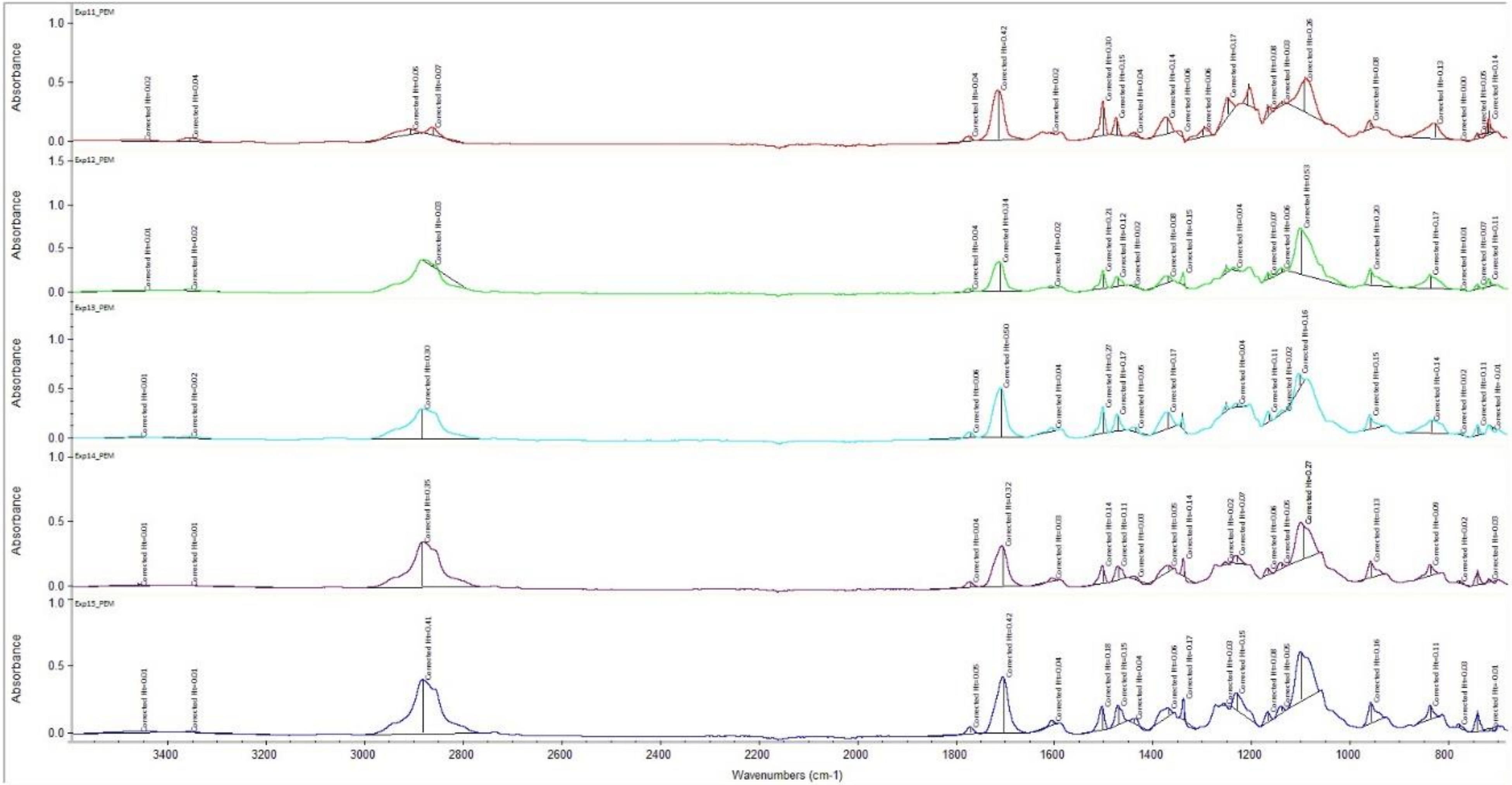

Supp. Fig 6. FTIR specta of PEMs 11-15 (used diamine is equal amounts of PDODA and AP6F) and height data of the peaks

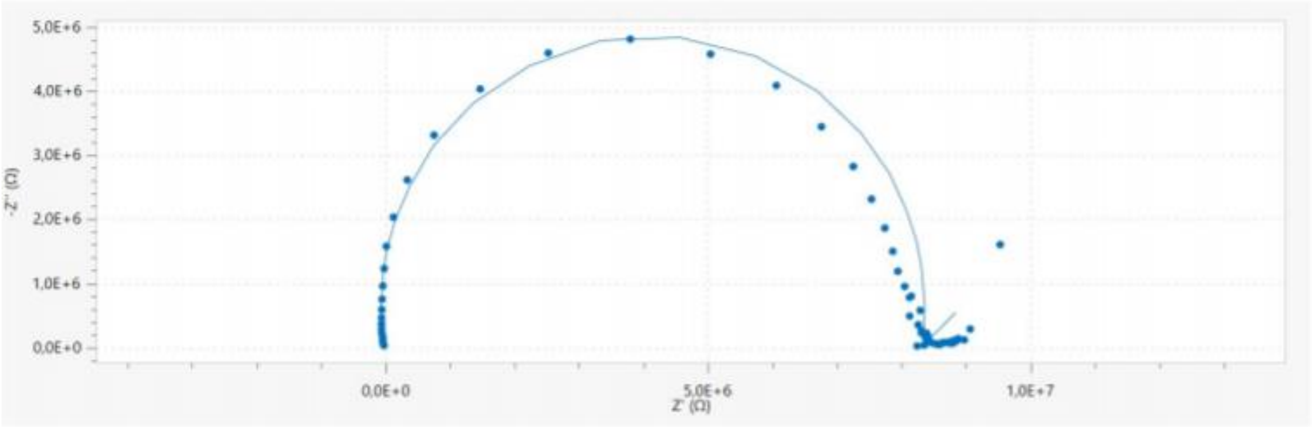

Supp. Fig 7. Nyquist plot and model fit for undoped membrane at 25°C (PEM_14 membrane was chosen)

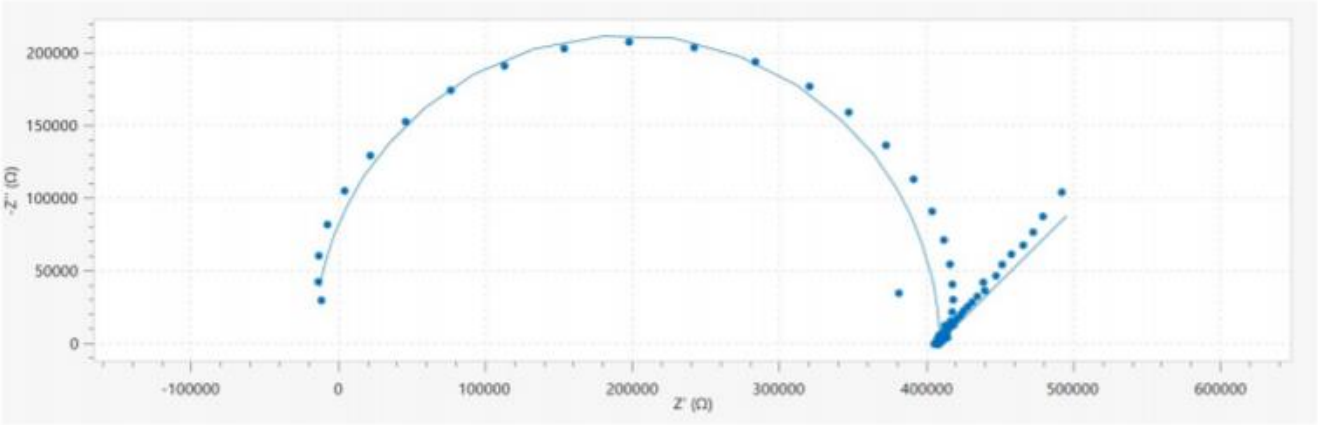

Supp. Fig 8. Nyquist plot and model fit for undoped membrane at 60°C (PEM_14 membrane was chosen)

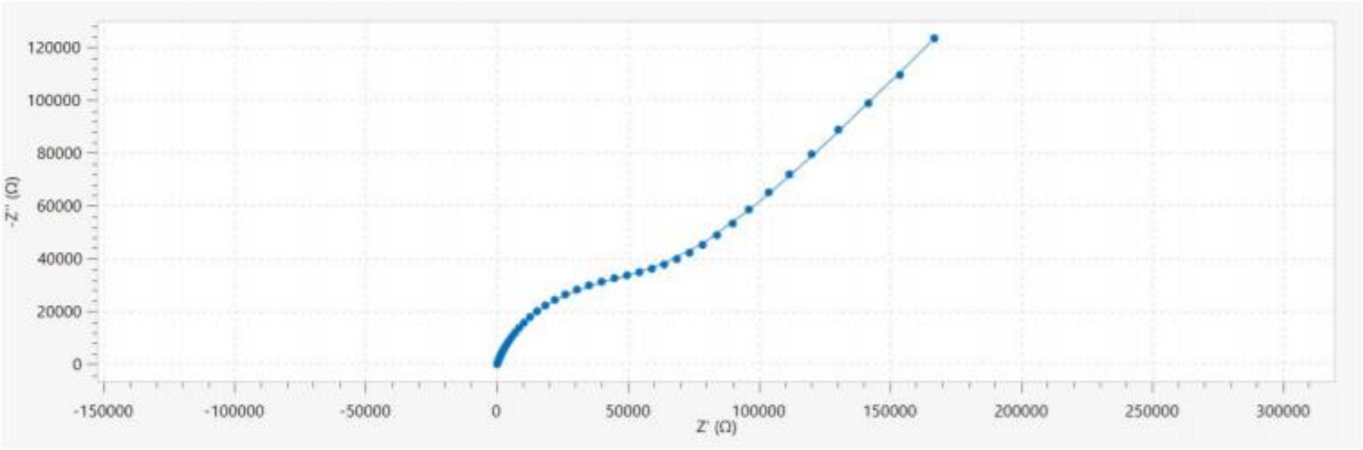

Supp. Fig 9. Nyquist plot and model fit for EAN doped membrane at 25°C (PEM_12 membrane was chosen)

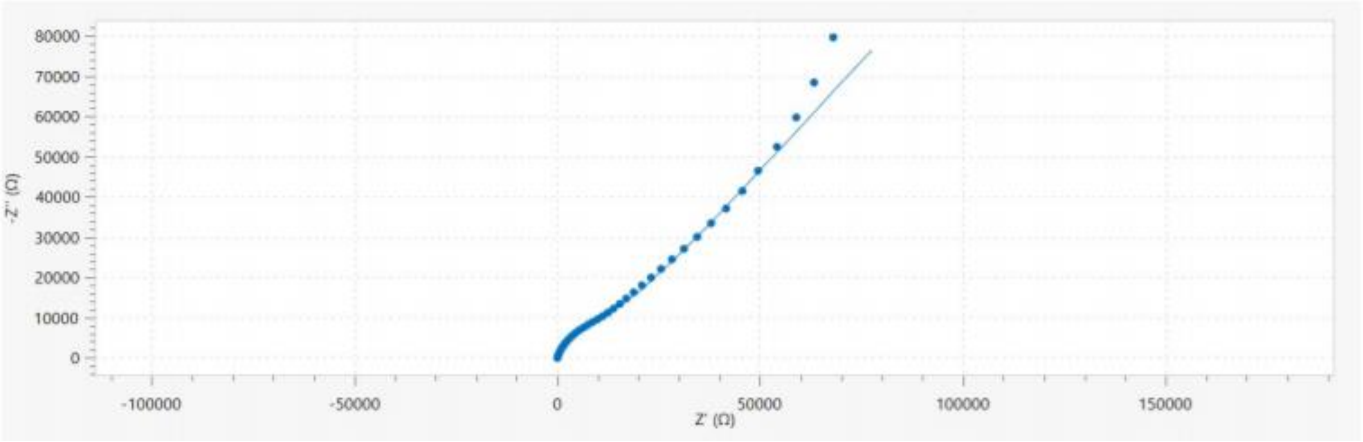

Supp. Fig 10. Nyquist plot and model fit for EAN doped membrane at 60°C (PEM_13 membrane was chosen)

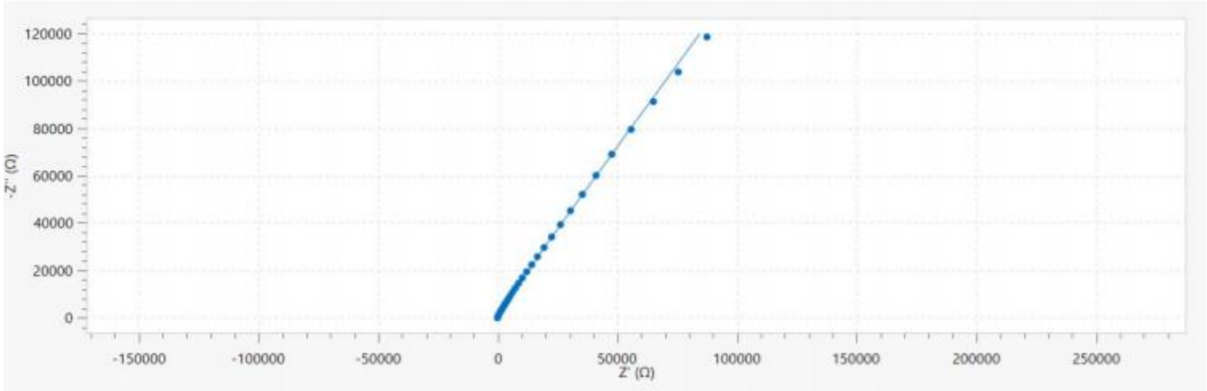

Supp. Fig 11. Nyquist plot and model fit for PAN doped membrane at 25°C (PEM_11 membrane was chosen)

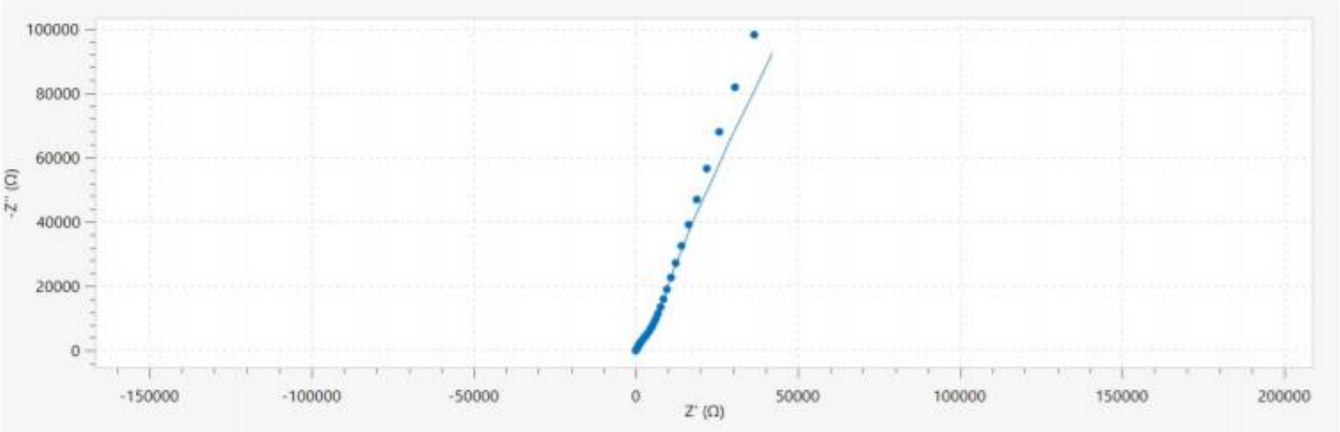

Supp. Fig 12. Nyquist plot and model fit for PAN doped membrane at 60°C (PEM_7 membrane was chosen)

Supplementary Table 1. Conductivity data for all the synthesized SBC membranes calculated by two methods; equivalent circuit fit and using real Z′ impedance value corresponding to lower imaginary Z′′ impedance at high frequency from the Nyquist Plot. All values are averages of three measurements. Selected Nyquist plots of the membranes are given as an example of each tested situation.

(PEG content is 42.1 wt% in all materials)							
**Membranes**	**Average Conductivities**	**25°C**			**60°C (Temperature Annealing for 1h)**		
		**UnDoped Membrane Film**	**Doped in EAN Ionic Liquid (1 day)**	**Doped in PAN Ionic Liquid (1 day)**	**UnDoped Membrane Film**	**Doped in EAN Ionic Liquid (1 day at 60°C)**	**Doped in PAN Ionic Liquid (1 day at 60°C)**
**PEM_1**	Average Conductivity from Electric Circuit Fit ($mScm^{-1}$)	**8.64E-02**	**75.56**	**83.01**	**6.30E-01**	**356.08**	**396.61**
	Average Conductivity from Nyquist Plot($mScm^{-1}$)	**8.76E-02**	**80.89**	**92.08**	**7.69E-01**	**333.99**	**377.10**
**PEM_2**	Average Conductivity from Electric Circuit Fit ($mScm^{-1}$)	**3.98E-02**	**84.59**	**91.57**	**5.87E-01**	**155.55**	**164.24**
	Average Conductivity from Nyquist Plot($mScm^{-1}$)	**3.96E-02**	**72.60**	**87.62**	**5.82E-01**	**151.55**	**168.27**
**PEM_3**	Average Conductivity from Electric Circuit Fit ($mScm^{-1}$)	**2.49E-02**	**59.72**	**131.59**	**6.18E-01**	**310.03**	**336.93**
	Average Conductivity from Nyquist Plot($mScm^{-1}$)	**2.54E-02**	**56.79**	**136.39**	**6.25E-01**	**319.07**	**355.96**
**PEM_4**	Average Conductivity from Electric Circuit Fit ($mScm^{-1}$)	**1.11E-02**	**60.61**	**146.86**	**3.09E-01**	**267.17**	**284.96**
	Average Conductivity from Nyquist Plot($mScm^{-1}$)	**1.11E-02**	**52.10**	**159.10**	**3.12E-01**	**285.06**	**300.62**
**PEM_5**	Average Conductivity from Electric Circuit Fit ($mScm^{-1}$)	**4.73E-03**	**19.46**	**31.75**	**8.51E-02**	**134.98**	**147.14**
	Average Conductivity from Nyquist Plot($mScm^{-1}$)	**4.43E-03**	**19.84**	**34.76**	**8.05E-02**	**127.60**	**153.68**

**PEM_6**	Average Conductivity from Electric Circuit Fit ($mScm^{-1}$)	**3.82E-03**	**116.39**	**135.82**	**3.91E-02**	**380.09**	**387.97**
	Average Conductivity from Nyquist Plot($mScm^{-1}$)	**3.88E-03**	**107.49**	**130.98**	**5.12E-02**	**372.92**	**385.33**
**PEM_7**	Average Conductivity from Electric Circuit Fit ($mScm^{-1}$)	**6.64E-04**	**94.84**	**120.44**	**3.39E-02**	**291.88**	**358.49**
	Average Conductivity from Nyquist Plot($mScm^{-1}$)	**5.58E-04**	**88.78**	**117.59**	**3.56E-02**	**283.33**	**344.78**
**PEM_8**	Average Conductivity from Electric Circuit Fit ($mScm^{-1}$)	**9.34E-04**	**84.64**	**93.47**	**2.10E-01**	**211.96**	**326.91**
	Average Conductivity from Nyquist Plot($mScm^{-1}$)	**8.92E-04**	**80.90**	**90.03**	**2.23E-01**	**205.51**	**321.46**
**PEM_9**	Average Conductivity from Electric Circuit Fit ($mScm^{-1}$)	**8.34E-04**	**29.07**	**100.83**	**1.49E-01**	**170.56**	**157.29**
	Average Conductivity from Nyquist Plot($mScm^{-1}$)	**7.39E-04**	**29.90**	**96.12**	**1.46E-01**	**168.40**	**154.97**
**PEM_10**	Average Conductivity from Electric Circuit Fit ($mScm^{-1}$)	**7.04E-04**	**8.92**	**70.48**	**1.92E-01**	**104.13**	**101.08**
	Average Conductivity from Nyquist Plot($mScm^{-1}$)	**6.58E-04**	**8.69**	**68.28**	**1.91E-01**	**104.45**	**98.43**
**PEM_11**	Average Conductivity from Electric Circuit Fit ($mScm^{-1}$)	**8.36E-02**	**106.79**	**116.20**	**5.09E-01**	**361.18**	**325.35**
	Average Conductivity from Nyquist Plot($mScm^{-1}$)	**8.26E-02**	**95.23**	**112.13**	**5.02E-01**	**351.09**	**361.54**
**PEM_12**	Average Conductivity from Electric Circuit Fit ($mScm^{-1}$)	**3.52E-02**	**88.94**	**135.37**	**2.11E-01**	**283.28**	**345.88**
	Average Conductivity from Nyquist Plot($mScm^{-1}$)	**3.56E-02**	**84.29**	**133.06**	**2.11E-01**	**261.42**	**323.37**
**PEM_13**	Average Conductivity from Electric Circuit Fit ($mScm^{-1}$)	**6.88E-02**	**53.92**	**71.15**	**7.23E-01**	**215.72**	**267.59**

	Average Conductivity from Nyquist Plot($mScm^{-1}$)	**7.48E-02**	**52.94**	**67.21**	**7.29E-01**	**207.48**	**267.27**
**PEM_14**	Average Conductivity from Electric Circuit Fit ($mScm^{-1}$)	**2.65E-03**	**39.54**	**74.52**	**5.50E-02**	**116.77**	**172.15**
	Average Conductivity from Nyquist Plot($mScm^{-1}$)	**2.68E-03**	**45.36**	**74.66**	**7.79E-02**	**114.09**	**174.64**
**PEM_15**	Average Conductivity from Electric Circuit Fit ($mScm^{-1}$)	**3.38E-03**	**35.09**	**32.98**	**7.74E-02**	**59.09**	**99.68**
	Average Conductivity from Nyquist Plot($mScm^{-1}$)	**3.36E-03**	**34.49**	**32.50**	**7.82E-02**	**60.90**	**106.52**
